# A pilot study of trained ICU doulas providing early psychological support to critically ill patients

**DOI:** 10.1186/s13054-021-03856-3

**Published:** 2021-12-20

**Authors:** Lioudmila V. Karnatovskaia, Katalin Varga, Alexander S. Niven, Phillip J. Schulte, Midhat Mujic, Ognjen Gajic, Brent A. Bauer, Matthew M. Clark, Roberto P. Benzo, Kemuel L. Philbrick

**Affiliations:** 1 Division of Pulmonary and Critical Care Medicine, Department of Medicine, Mayo Clinic, 200 First Street SW, Rochester, MN 55905 USA; 2 Affective Psychology Department, Eötvös Loránd University, Budapest, Hungary; 3 Biomedical Statistics and Informatics, Mayo Clinic, Rochester, MN USA; 4 Anesthesia Clinical Research Unit, Mayo Clinic, Rochester, MN USA; 5 Department of Psychiatry and Psychology, Mayo Clinic, Rochester, MN USA

**Keywords:** Therapeutic suggestion, Positive suggestion, Critically ill, Doula, Psychological support, PTSD, Anxiety, Depression

## Abstract

**Background:**

Over a third of critical illness survivors suffer from mental health problems following hospitalization. Memories of delusional experiences are a major risk factor. In this project, ICU doulas delivered a unique positive suggestion intervention targeting the vulnerable time period during critical illness when these memories are formed.

**Methods:**

Adult critically ill patients were recruited for this single-arm, prospective pilot study. These ICU patients received a positive suggestion intervention daily during their ICU stay in parallel with their medical treatment. The intervention was designed to be delivered over a minimum of two sessions. Feasibility was defined as intervention delivery on ≥ 70% of ICU days after patient enrollment. As a secondary analysis, psychometric questionnaires were compared to those of a historic control cohort of patients receiving standard care in the ICU using adjusted linear regression models.

**Results:**

Of the 97 patients who received the intervention and were alive at the end of their ICU course, 54 were excluded from analyses mostly for having received only one session because of a short ICU length of stay of < 2 days, transitioning to comfort care or not wanting to answer the study questionnaires. Forty-three patients who completed 2 or more sessions of the positive therapeutic suggestion intervention provided by two trained ICU doulas received it for a median of 4 days (IQR 3, 5), with each session lasting for a median of 20 min (IQR 14, 25). The intervention was delivered on 71% of days, meeting our pre-determined feasibility goal. Compared to historical controls (*N* = 299), patients receiving the intervention had higher severity of illness and longer length of stay. When adjusted for baseline differences, patients both with and without mechanical ventilation who received the intervention scored lower on the Hospital Anxiety and Depression Scale (HADS)—Depression subscale. The intervention was also associated with reduced HADS-Anxiety subscale among ventilated patients.

**Conclusions:**

Positive therapeutic suggestion delivered by ICU doulas is feasible in the ICU setting. A randomized trial is warranted to better delineate the role that positive suggestion and ICU doulas may play in ongoing interprofessional efforts to humanize critical care medicine.

The study was registered on clinicaltrials.gov (NCT03736954) on 03/14/2018 prior to the first patient enrollment https://clinicaltrials.gov/ct2/show/NCT03736954?cond=ICU+Doulas+Providing+Psychological+Support&draw=2&rank=1.

**Supplementary Information:**

The online version contains supplementary material available at 10.1186/s13054-021-03856-3.

## Background

Admission to the intensive care unit (ICU) can be a traumatic experience, and over a third of critical illness survivors struggle with post-intensive care syndrome or PICS following hospitalization characterized by symptoms of anxiety, depression, and post-traumatic stress disorder (PTSD) as well as cognitive and physical impairments [1–5]. There have been significant advances in the reduction of cognitive impairment via ICU delirium prevention strategies [6], and early mobility in the ICU has become a commonplace intervention to mitigate physical impairment [7]. A variety of approaches have been tried to address PICS-related mental health impairments including ICU diaries [8, 9], nurse-led interventions [10, 11], music therapy [12], and psychotherapist-provided interventions [13–15] with various degrees of success. While psychotherapist-based interventions appear to provide the most promising preliminary data [16], availability of psychotherapists who can join the ICU practice is very limited. As of now, there are no published evidence-based interventions available to effectively prevent or treat mental health impairment in critical illness survivors.

Memories of frightening and delusional experiences are a major potentially modifiable risk factor [1, 17]. Research on fear memory development has demonstrated that introducing mitigating information about a traumatic event following memory formation and recall can modify its emotional spectrum [18]. One potential approach could involve interfering with the initial process of fear conditioning, by providing positive reframing of the hospital course to the patient in real time. For the critically ill, this would mean introducing psychological support as soon as feasible following ICU admission and providing it in parallel with ongoing medical care. However, most conventional forms of psychotherapy require active patient participation, limiting their application during these early stages of fear memory formation.

A therapeutic approach called Psychological Support Based on Positive Suggestions (PSBPS) is a form of suggestion therapy adapted to communicate with the critically ill [19]. PSBPS can involve both unidirectional and bidirectional communication and therefore can be performed regardless of patient participation level, as semantic processing continues in altered states of consciousness [20–22]. In preliminary studies, PSBPS by trained clinical psychologists has been associated with earlier ventilator liberation and decreased sedative and analgesic medication requirements [23–25], with early initiation of positive reframing crucial for these favorable outcomes. Its effect on mental health outcomes has not been investigated.

Unfortunately, clinical psychologists are rarely present in an ICU setting. A survey of all practicing psychologists belonging to the American Psychological Association and American Academy of Clinical Neuropsychology identified only 84 individuals who reported ever practicing in the ICU setting, most commonly performing cognitive assessments or capacity evaluations [26]. Therefore, we explored training alternate providers to perform early psychological support in the ICU setting. We identified doulas as the most viable candidates given the spectrum of emotional support they already provide to women in labor [27, 28], which is similar to what is needed in PSBPS.

The aim of this study was to evaluate the feasibility of delivering psychological support to patients in the ICU setting using doulas. We hypothesized that once trained for this role, doulas would be able to consistently provide PSBPS to the critically ill.

## Methods

### Study design and setting

We performed a prospective interventional cohort pilot project targeting patients admitted to a medical ICU in a tertiary care center between November 2018 and May 2019. Psychiatric and cognitive outcomes of the study patients were compared to those of historical controls [29]. These historical controls were surveyed to determine baseline mood impairment across a sample of ICU cohorts in our institution in comparison with national benchmarks.

### Participants

Inclusion criteria were adults (age ≥ 18) admitted to the medical ICU, requiring vasopressor support and/or mechanical ventilation and expected to stay ≥ 48 h. Exclusion criteria included a history of dementia, acute alcohol/substance abuse or withdrawal, admission following a suicide attempt, psychotic disorders such as schizophrenia, patients with a recent ICU admission (within 6 months), incarceration, severe metabolic encephalopathy, status epilepticus, non-English speaking, and patients not expected to survive their hospital stay or who were receiving only symptomatic treatment at the end of life. Delirium was not an exclusion criterion. The rationale for these exclusion criteria included identifying patients who would be able to answer follow-up study questionnaires and whose impressions would reflect their current stay and the intervention they received without the potential confounding of prior recent ICU experiences. Eligible patients were systematically screened daily Monday through Friday using electronic medical record review; those meeting inclusion criteria were approached once feasible following ICU arrival. Informed verbal consent was obtained by a study coordinator from a legally authorized representative in most cases. Once the patient was able to interact, continued study participation was verified with them as well.

### Intervention

Two doulas with combined 15-year practice experience underwent an extensive ICU training program to deliver the intervention [30]. In brief, training included three key learning objectives: (1) orientation to the structure and workflow of the ICU, (2) development of a fundamental understanding of common medical conditions specific to the ICU practice, and (3) delivery of PSBPS in the ICU setting [30]. Moreover, the doulas learned about the aspects of the ICU stay associated with formation of negative memories; reframing of those aspects was an integral part of their interventional approach.

We have previously collaborated with our colleagues in Hungary to design a PSBPS intervention script that could be used by non-psychologists and adapted to each clinical scenario [31]. The intervention consisted of 3 phases (Table 1).

**Table 1:** Psychological support based on positive suggestion intervention outline

Main phases of psychological support based on positive suggestion
Phase 1—Inform the patient about their ICU care and the nature of this treatment (daily)
1. Information/environment—positive summary of medical course
a. Introduce yourself and explain where patient is now
b. Explain briefly what happened/provide treatment update
c. Explain elements of treatment
1. Ventilation aspects of treatment—simple phrases/explanations
2. Procedural aspects—positive reframing/preframing based on each case
2. Reduction of psychological stressors
a. Non-verbal suggestion as appropriate—hand holding, etc.
Phase 2. Involvement of patient actively in the treatment process (once awake)
3. Positive commands/regaining control
a. Encourage patient to communicate with caregivers (even when they cannot speak) and to express their wishes/questions/requests
b. Ask to move a hand, make a fist, wiggle toes; provide positive feedback. Engage nurse. Encourage participation with physical therapy to provide patient with greater functional independence once transferred out of the ICU
c. Stimulate as many sensory channels as possible
d. Offer choices to give patient the experience of being in control/having the opportunity to participate in treatment
4. Optimistic future orientation/setting goals
a. Based on values/hobbies once patient can speak/hobbies known. Encourage patient to describe a positive experience from the past (thereby giving patient the opportunity to experience it again)
b. Set realistic aims together—today: get up; tomorrow: walk to the door, etc.
Phase 3. Debrief (once ready for discharge from ICU)
5. Have patient explain how they saw:
a. What happened to them
b. What they understood about their condition
c. How was being in the ICU for them
d. Are there any questions? Anything to clarify?

Each session started with Phase 1 which included an introduction, an update on the ICU course, and an explanation of both treatments and procedures continued from the previous day (such as mechanical ventilation) and those anticipated in the near future. Aspects of the ICU environment frequently associated with negative memories, such as noise, lights, blood draws, and imaging, were reframed as signals to the team to keep the patient safe and continue optimal treatment. Events were reframed and planned procedures pre-framed in a neutral or positive manner (for example, a noxious stimulus such as endotracheal tube suctioning was explained as a way to clear the mucus that we would normally cough up; a urinary catheter as a way to help the body get rid of urine cleanly). Patients were reassured that the ICU was the safest place in their current condition. This communication was performed regardless of the patient’s sedation status, presence or absence of delirium, and ability to communicate. When appropriate, this included the doulas holding the patient’s hand.

Phase 2 started once a patient was no longer sedated and was able to communicate. At this point a bidirectional connection was established by asking the patient to follow simple commands. Patients were encouraged to assume a more active role in their recovery; individual goals were established with an optimistic, future orientation (e.g., extubation was outlined as a goal, when they are strong enough for physiological breathing).

Phase 3 started once the patient was stable for discharge from the ICU. This phase consisted of debriefing, focusing on explanations of what to expect after transfer to the ward and during subsequent recovery, an exploration of the patient’s fears and concerns related to their ICU stay, and normalizing their experience.

### Outcome measures

The primary outcome was feasibility, defined as delivering the intervention on ≥ 70% of the days for enrolled patients while they were in the ICU. Other metrics included session duration, frequency, and interruptions or patient/family refusal. Secondary outcomes examined the association between the intervention and accepted measures of anxiety, depression, and post-traumatic stress disorder (PTSD). Patients were approached by research staff blinded to the intervention to complete psycho-cognitive outcome measures within 96 h of transfer out of the ICU.

The Hospital Anxiety and Depression Scale (HADS), previously validated in a critically ill population, is composed of seven items measuring general anxiety and depressive symptoms each rated on a four-point scale (0–3) [32]. A score of ≥ 8 is considered symptomatic; the study coordinator was required to notify the primary team if they found a patient had a HADS-Depression (HADS-D) score ≥ 11. Post-traumatic stress disorder (PTSD) symptoms were assessed using the Impact of Event Scale-Revised (IES-R) [33]. The IES-R consists of 22 items with each rated on a five-point scale (0–4), with values of ≥ 1.6 having 100% sensitivity and 85% specificity for detecting PTSD [33]. For the intervention trials measuring intervention efficacy for the patient, 2.0–2.5 is an accepted threshold range for the HADS-A and HADS-D subscales, and 0.2 is the minimum necessary difference for the IES-R [34].

Cognitive function was assessed using Montreal Cognitive Assessment (MoCA)-Blind. The MoCA is a tool routinely used by occupational therapists at our institution to screen for mild cognitive impairment [35]. We used the MoCA-Blind as this instrument can be administered by telephone [36]. The MoCA-Blind is a practical tool to efficiently assess current cognitive dysfunction in a critically ill or recently critically ill population, with a total score ≥ 18 considered normal [37]. Use of the above screening tools has been recommended by the Society of Critical Care Medicine for prediction and identification of long-term impairments after critical illness [38].

### Historical controls

We compared the results of this intervention with data available from a prior cross-sectional prospective observational study of 299 patients admitted to Mayo Clinic ICUs between January and November of 2016. These patients were enrolled using similar exclusion criteria as the patients recruited for this intervention and received standard medical care in addition to completing HADS, IES-R, and MoCA-Blind assessments within 96 h of ICU discharge while still in the hospital. Clinical data and outcomes were previously extracted from the electronic medical record.

### Statistical analyses

The primary outcomes relate to feasibility; secondary outcomes provide pilot psychometric data among intervention patients in the single arm intervention trial. When compared to historical controls, we first compared demographics and ICU admission characteristics of the intervention and historic control groups using median (25th, 75th) percentiles and Wilcoxon rank-sum tests for continuous variables; categorical variables were compared with percentage and Pearson Chi-square test or Fisher’s exact test. Secondary outcomes including HADS-A, HADS-D, IES-R subscores, and MoCA-Blind were described and compared similarly.

Linear regression models were used to assess the association between intervention and each secondary outcome, adjusted for pre-specified covariates including patient age, sex, history of depression, history of anxiety, and use of mechanical ventilation. We assessed the interaction between intervention and mechanical ventilation to evaluate whether there was evidence that the association between the doula intervention and outcomes differed according to whether patients were on mechanical ventilation. When interaction *p* values were < 0.10, we report the association between intervention and outcome separately for ventilated and non-ventilated subjects. Otherwise, the interaction was removed, and we report the overall association between intervention and outcomes. Results are reported as the estimated mean difference in outcome between intervention-treated patients and standard care-treated patients, with 95% confidence intervals (CIs) and *p* values. Residual diagnostics including assessment of normality of residuals and homoscedasticity of residuals were visually assessed in plots (Q-Q plot, residuals vs. predicted, etc.); no violations were suggested.

A sensitivity analysis was performed restricting the historic standard care-treated patients to those admitted to the MICU as the historic controls included patients from six adult ICUs. The primary aim of the current study was to assess feasibility of the intervention and to obtain pilot data. As such, outcome analyses are exploratory, and no adjustment is made for analyzing multiple outcomes.

## Results

**Fig. 1:**
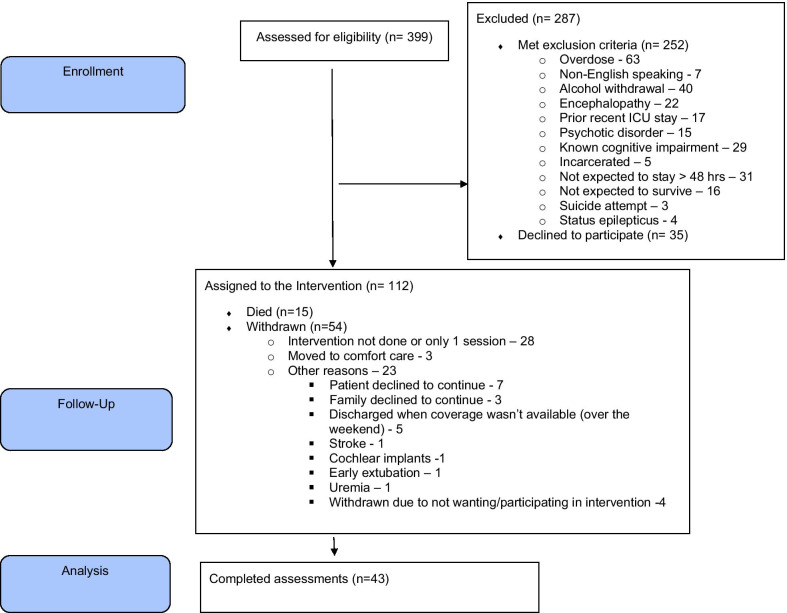
Flow diagram describing study recruitment

Forty-three patients (see Fig. 1) received the PSBPS intervention provided by two trained ICU doulas for a median of 4 days (3, 5) with each session lasting a median of 20 min (14, 25). Of the 277 total days patients were enrolled, the intervention was delivered on 198 days (71%) meeting our pre-determined feasibility goal. The main reasons for failing to deliver the intervention were doula absence (due to competing responsibilities and a particularly severe winter season with multiple heavy snowstorms), followed by procedures, nursing requests not to disturb the patient while sleeping, and family request not to interrupt their visit with the patient. Notably, of the 112 enrolled patients only 43 completed final study assessments. Of the remaining 54 who were alive at the end of their ICU course, the main reason identified for not gathering study assessment was cases when the intervention was received only once due to an ICU length of stay of less than 2 days (*N* = 28). It was a priori decided that only the patients who received 2 or more study intervention sessions would subsequently be classified as “study completers” given that the intervention was not designed to be a single stand-alone session. As the ICU doulas were not embedded into the ICU and were continuing their active doula practice, sometimes they were not able to make it to the hospital and see the patient on the day of their ICU admission if the patient was admitted late in the afternoon. Therefore, sometimes they would see the patient the morning of the next day, and by the afternoon some patients would already be extubated and transferred to the hospital ward.

Additionally, 7 patients did not wish to complete the study questionnaires and declined further study participation once they were approached by the study team to complete the questionnaires; main reasons were fatigue and difficulty concentrating. In three instances, families declined—two cases of not wishing to burden their loved ones with the study assessments, and in one instance interpreting the intervention as the doulas “talking down to the patient,” saying that the patient was very independent at baseline and would never want someone to explain things to her in that much detail. Notably, that patient later tracked down the ICU doulas and thanked them profusely for explaining things to her in a simple and easy to understand way and for filling in the blanks, so she knew what was happening during the weeks she stayed mechanically ventilated.

Patients receiving the intervention had a median age of 67 (58, 74), and 58% were male (Table 2). Compared to historical controls, intervention patients more frequently had a history of anxiety (49%) or depression (49%); they had higher median APACHE 2 score of 91 (64, 106); there were no differences in the Charlson comorbidity score. The historical patient cohort (*n* = 299) had a median age of 63 (54, 71), and 57% were male. The rate of mechanical ventilation in the ICU was higher in the intervention group (67% vs 46%, *p* = 0.01). In comparing data between only historical MICU patients and the intervention patients, results were similar (Additional file 1: Table S1).

**Table 2:** Comparison of demographics and ICU characteristics between treatment and control patients

Demographic variables	Historic controls (*N* = 299)	PSBPS group (*N* = 43)	*p* value
Age	63 (54, 71)	67 (58, 74)	0.197
Male sex	170 (57%)	25 (58%)	0.874
ICU type			< 0.001
Cardiac MICU	50 (17%)	0 (0%)	
Cardiothoracic SICU	50 (17%)	0 (0%)	
Heme-onc/transplant MICU/SICU	49 (16%)	0 (0%)	
MICU	50 (17%)	43 (100%)	
SICU	50 (17%)	0 (0%)	
Trauma SICU	50 (17%)	0 (0%)	
Apache score	41 (30, 56)	91 (64, 106)	< 0.001
Mechanical ventilation	139 (46%)	29 (67%)	0.010
MV days	1.1 (0.3, 2.7)	2.0 (1.4, 4.1)	0.002
ICU LOS	3.1 (2.5, 5.0)	4.5 (2.9, 7.3)	0.007
Anxiety	92 (31%)	21 (49%)	0.019
Depression	79 (26%)	21 (49%)	0.003
Charlson score	5 (3, 7)	5 (4, 8)	0.551

Among patients receiving the intervention, median (25th, 75th) MoCA-Blind total score was 16 (13, 18), while historical controls had higher MoCA-Blind of 17 (15, 19) (*p* = 0.03) (Table 3). HADS-D and HADS-A scores were similar between the groups, with scores among the intervention patients 6 (3, 7) and 6 (4, 9) for depression and anxiety, respectively.

**Table 3:** Univariable comparisons of outcomes between treatment and control patients

	Historic controls (*N* = 299)	PSBPS group (*N* = 43)	Total (*N* = 342)	*p* value
MoCA-BLIND total (0–22)	17 (15, 19)	16 (13, 18)	17 (14, 19)	0.030
HADS depression score (0–21)	6 (3, 9)	6 (3, 7)	6 (3, 9)	0.320
HADS anxiety score (0–21)	7 (4, 10)	6 (4, 9)	7 (4, 9)	0.284
IES-R intrusion score (0–4)	0.9 (0.3, 1.7)	0.7 (0.3, 1.6)	0.9 (0.3, 1.7)	0.388
IES-R avoidance score (0–4)	0.8 (0.3, 1.5)	0.7 (0.3, 1.7)	0.8 (0.3, 1.5)	0.837
IES-R hyperarousal score (0–4)	1.0 (0.3, 1.7)	0.8 (0.2, 1.6)	1.0 (0.3, 1.7)	0.755

Following multivariable adjustment, there was no evidence to suggest a difference in MoCA-Blind by intervention group (adjusted estimated difference = − 0.7, 95%CI = − 1.8, 0.3, *p* = 0.18) (Table 4). In multivariable models, there was evidence of an interaction between mechanical ventilation and treatment group for the outcome of HADS-A (interaction *p* = 0.10). Among those without mechanical ventilation, doula intervention was not associated with different HADS-A outcomes compared to historical controls; however, among those on mechanical ventilation, doula intervention was associated with reduced HADS-A score (estimated adjusted difference = − 2.0, 95%CI = − 3.6, − 0.3, *p* = 0.019). Overall, there was evidence of reduced HADS-D associated with doulas intervention among the entire sample (estimate = − 1.3, 95%CI = − 2.6, 0.0, *p* = 0.049). Numerical point-estimates in adjusted models for IES-R subscores were not statistically significant.

**Table 4:** Summary of treatment association from multivariable analyses

Outcome	Estimate (95% CI)	*p* value
MoCA-BLIND total (0–22)	− 0.7 (− 1.8, 0.3)	0.18
HADS depression score (0–21)	− 1.3 (− 2.6, − 0.0)	0.049
IES-R intrusion score (0–4)	− 0.26 (− 0.57, 0.04)	0.087
IES-R avoidance score (0–4)	− 0.08 (− 0.39, 0.22)	0.60
IES-R hyperarousal score (0–4)	− 0.18 (− 0.51, 0.14)	0.26
HADS anxiety score (0–21)		
Among no mechanical ventilation	0.4 (− 1.9, 2.7)	0.73
Among mechanical ventilation	− 2.0 (− 3.6, − 0.3)	0.019

When the historical cohort were limited to the subgroup admitted to the MICU (*n* = 50) there were still differences in baseline APACHE score (median (25th, 75th) = 38 (28, 44) among historic MICU cohort vs 91 (64, 106) among doulas intervention, *p* < 0.001. There were also differences in use of mechanical ventilation (24% vs 67%, *p* < 0.001) and ICU length of stay (3.1 (2.7, 4.3) vs 4.5 (2.9, 7.3), *p* = 0.020. History of depression was less common in this subgroup of historic controls (28% vs 49%, *p* = 0.039) compared to the doula intervention. In multivariable models, there were no statistically significant differences between the doula intervention group and MICU historical cohort; while confidence intervals were wide, point estimates favored the ICU doulas group (Table 5).

**Table 5:** Summary of treatment association from multivariable analyses, in sensitivity analysis using MICU controls

Outcome	Estimate (95% CI)	*p* value
MoCA-BLIND total (0–22)	− 0.2 (− 1.7, 1.7)	0.98
HADS depression score (0–21)	− 0.8 (− 2.5, 0.9)	0.35
HADS anxiety score (0–21)	− 1.0 (− 3.0, 0.9)	0.29
IES-R intrusion score (0–4)	− 0.31 (− 0.74, 0.11)	0.15
IES-R avoidance score (0–4)	− 0.05 (− 0.53, 0.42)	0.82
IES-R hyperarousal score (0–4)	− 0.31 (− 0.78, 0.16)	0.19

## Discussion

This single-arm pilot project demonstrated that trained doulas could feasibly and successfully deliver a program based on positive suggestion in the ICU setting to critically ill patients. The large number of patients who received one intervention session only and were therefore excluded from analyses represent the standard of practice of that time when average length of stay was very short. For people with longer length of stay who received two or more sessions of the intervention, psychometric assessments suggest that this intervention is associated with lower subsequent symptoms of anxiety and depression in critical illness survivors. Given the study design, however, the observed differences are hypothesis generating only at this point and causation cannot be assigned.

Previously, we examined feasibility of PSBPS being performed by trained intensivists. While the study was not powered to estimate effects on mental health outcomes, we did observe that the intervention was associated with the lower estimated odds for anxiety compared to the historical cohort (OR = 0.38, 95% CI = 0.07, 1.8) [31]. While it was feasible to conduct the intervention when the study physician was on service, it was ultimately deemed to be impractical to advocate training already busy clinicians to deliver full PSBPS sessions in addition to their already busy practice. We hope to see in the near future licensed health care providers (physicians, nurse practitioners, and nurses) receiving basic professional training in PSBPS-like communication with the critically ill, as the provider’s “voice” and choice of words would likely have an even greater impact on the patient.

Our findings offer a novel and potentially less expensive solution to deliver a psychotherapeutic intervention in the ICU. German investigators reported training psychotherapists to provide ICU patients receiving non-invasive ventilation with hypnotic suggestions of relaxation and safety. Similar to the results of the current study, they found lower patient anxiety levels following the intervention [13]. An American institution published a pilot randomized controlled trial of cognitive behavioral therapy, including 4 individual, in-person, bedside psychotherapy sessions conducted by trained psychology fellows and lasting 60 to 90 min each. Participants in the intervention group had improved depression and PTSD scores compared to their pre-treatment baseline [15]. In another study from same center, a clinical psychologist embedded in the medical ICU practice provided consultation, assessment and treatment for anxiety to patients in acute respiratory failure. Following completion of 6 sessions of about 30 min duration each, the authors reported significant after-session decreases in the visual analog scale anxiety score compared to baseline pre-intervention data [14]. Clinical psychologists also conducted educational sessions for ICU patients in Italy with investigators reporting a significant reduction in IES-R scores and a trend toward lower HADS scores at 12 months after hospital discharge in trauma patients compared to historical controls [16]. Although these results are intriguing, availability of trained psychotherapists, costs of employing them full time in the ICU setting, and duration of their training would limit generalizability of these findings. For that reason, we explored alternative options by training doulas to provide a supportive intervention in the ICU setting. In contrast to the limited number of practicing psychotherapists who can join the ICU practice, there are many doulas working nationwide; doula training can be completed in a significantly shorter time frame; additional training to work in the ICU setting can be accomplished in a month, and the cost associated with their salaries is also lower.

Other interventions to improve psychological outcomes include ICU diaries. A Cochrane systematic review found minimal evidence from randomized controlled trials of the benefits of the diaries for the ICU patients [8]. A more recent meta-analysis reported a decrease in anxiety and depression metrics without an effect on PTSD [9], while another meta-analysis published in the same year concluded that the available evidence on the benefit of diaries was “thin” [39]. A meta-analysis of music therapy trials described a consistent reduction in anxiety despite heterogeneity of trial designs [12]. Nurse-led consultations failed to reduce PTSD symptoms in patients following ICU discharge [11]. A recent multi-center nurse-led preventive psychological intervention, initiated in the ICU, failed to reduce patient-reported mental health symptom severity at 6 months [10]. A single-center randomized trial of a mindfulness app following hospital discharge compared to a therapist-led telephone-based mindfulness program demonstrated similar reduction in depression scores between the two interventions [40]. Clearly more research is needed to identify strategies to reduce the onset of mental health problems following an ICU hospitalization.

Our study is unique as the PSBPS was administered to patients who were sedated and often mechanically ventilated, thus precluding us from obtaining baseline psychometric data for comparison. Although not powered to detect differences in psychiatric outcomes, preliminary historic control data comparison is nonetheless encouraging. This study has several important limitations. Patients were not prospectively randomized to intervention or control conditions, and therefore causative statements cannot be made. A large number of patients were excluded from analyses due to having received only one intervention session. The study was also not powered to detect differences in secondary outcomes of depression, anxiety, and PTSD. The risk of confounding bias in comparison with the historical control reference set is significant, thereby limiting conclusions that can be drawn from such comparison to anything but an association between this intervention and reduced anxiety and depression, which is hypothesis generating and merits further study. Session duration is also overall shorter than reported for targeted interventions performed by therapists. Doulas were also not able to be present in the ICU daily due to competing responsibilities and winter storms. Finally, our medical population is primarily white, so how these findings might apply to more diverse or underserved populations is unknown.

In summary, trained ICU doulas were readily accepted and delivery of positive therapeutic suggestion was feasible in the ICU setting. The mood data are hypothesis generating only at this point, but these findings do support further study in a randomized controlled efficacy trial of the intervention. As the HADS score ≥ 8 is considered symptomatic, a future trial would need to assess whether we would observe such a difference between the groups, because if the post-intervention scores fall below the symptomatic threshold, the intervention would demonstrate clinical significance for the patient as well. If PSBPS is determined to be effective, then training additional doulas to work in the ICU setting may be a faster and potentially less expensive approach, compared to adding a clinical psychologist to the ICU team.

## Conclusions

Positive therapeutic suggestion performed in parallel with medical treatment in the ICU is a feasible intervention which warrants further exploration on its impact on psychiatric morbidity among critical illness survivors and humanizing critical care. A larger randomized clinical trial is warranted to assess efficacy of PSBPS as performed by trained ICU doulas.

## Supplementary Information

**Additional file 1.** Comparison of demographics and ICU characteristics between treatment and control patients among MICU.

## Data Availability

Available upon request.
