# Supplementary material for: A pilot study of trained ICU doulas providing early psychological support to critically ill patients
Source: Crit Care. 2021 Dec 20;25:446. doi: 10.1186/s13054-021-03856-3 (PMC8691072; doi:10.1186/s13054-021-03856-3)
Supplement: Supplementary file 1 — Additional file 1. Comparison of demographics and ICU characteristics between treatment and control patients among MICU. [file 13054_2021_3856_MOESM1_ESM.docx]

Table 2S. Comparison of demographics and ICU characteristics between treatment and control patients among MICU

| **Demographic Variables** | **Historic controls (N=50)** | **PSBPS group (N=43)** | **p value** |
| --- | --- | --- | --- |
| **Age** | 65 (58, 75) | 67 (58, 74) | 0.923 |
| **Male Sex** | 26 (52%) | 25 (58%) | 0.553 |
| **ICU Type** |  |  |  |
| MICU | 50 (100%) | 43 (100%) |  |
| **Apache score** | 38 (28, 44) | 91 (64, 106) | < 0.001 |
| **Mechanical ventilation** | 12 (24%) | 29 (67%) | < 0.001 |
| **MV Days** | 1.7 (1.1, 1.9) | 2.0 (1.4, 4.1) | 0.069 |
| **ICU LOS** | 3.1 (2.7, 4.3) | 4.5 (2.9, 7.3) | 0.020 |
| **Anxiety** | 20 (40%) | 21 (49%) | 0.392 |
| **Depression** | 14 (28%) | 21 (49%) | 0.039 |
| **Charlson Score** | 4 (3, 7) | 5 (4, 8) | 0.855 |
